# An Introduction to Dermatology

**Published:** 1912-02

**Authors:** 


**An Introduction to Dermatology,** by **Norman Walker,** M.D., F.R.C.P., Physician for Diseases of the Skin, The Royal Infirmary, Edinburgh. Fifth edition, 1911. Published by William Wood & Co., New York. 346 pages, 43 colored plates and 79 illustrations.

In volume 39, page 306, of this journal, about twelve years ago, in commenting upon the first edition of Walker's Introduction to Dermatology, we took occasion to commend to the attention of physicians for whom the author primarily intended his work and who undoubtedly gained considerable information from it about the subjects of which it treated; but it is very doubtful if the knowledge acquired therefrom has been of much use to the student as was stated in reviewing this edition for it conveyed so much of the then latest information upon disputed points concerning skin diseases, that it probably led the mere student to dangerous conclusions. Nevertheless, Dr. Walker's book became a standard, not alone in England, but in America as well.

The fifth edition is a marked improvement over the first, the author making it more practical; the features that appeared somewhat too positive in former editions have been modified but we cannot deny the verity and originality which then characterized his exposition of them. The language is condensed, but never to the point of obscurity. The author has borrowed from medical sources, but the work is essentially an expression of personal observation. The topics considered, although briefly disposed of in some instances, are numerous and varied. For instance, syphilis only receives a few pages and as stated in the previous review, it would have been better had it been omitted, although the many criticisms mentioned in the first review have been heeded and the book shaped along lines which are permanent. This edition at least looks far into the future and is certain from its excellence to be long lived, because of the improvements due to repeated revision.

The illustrations are for the most part of a practical kind; some are merely outlined in black and white, some are photographs from life and appeared in the first edition, and some are now colored plates from casts which were beautiful examples of accurate representation of cutaneous eruptions which would be difficult to improve. The text, paper and binding are of a high class; the physician, even the dermatologist will find this volume a distinct addition to the already formidable list of dermatologic text-books. G.W.W.

